# Selective Glenohumeral external rotation deficit – sequelae of post-ORIF deltoid adhesions after treatment of the proximal humerus fracture

**DOI:** 10.1186/s12891-020-03634-2

**Published:** 2020-09-22

**Authors:** Michał Waszczykowski, Jarosław Fabiś

**Affiliations:** 1 Department of Arthroscopy, Minimally Invasive Surgery and Sports Traumatology, Medical University of Lodz, Lodz, Poland, ul. Żeromskiego 113, 90-549 Lodz, Poland; 2 FMC Medical Center, 9A Piłsudskiego, Lodz, Poland

**Keywords:** Proximal humerus fracture, Arthroscopic plate removal, Selective glenohumeral external rotation deficit, Deltoid adhesion, Deltohumeral space

## Abstract

**Background:**

The deltopectoral approach is commonly used for plate stabilization of proximal humerus fracture. Although adhesions between the deltoid, plate, and humerus are common sequelae of plate ORIF, little is known about their effect on the range of movement and a function of the shoulder. To confirm their impact, the preoperative and intraoperative evaluation of the range of motion (ROM) was measured during the sequential arthroscopic release of adhesions, with special regard to external rotation. Postoperative ROM and subjective shoulder function were also evaluated.

**Methods:**

Eighteen patients treated with ORIF of the proximal humerus were scheduled to the unified arthroscopic procedures comprising sequential limited subacromial bursectomy, removal of the adhesions between the deltoid, plate, and humerus, as well as the plate removal. The ROM of the operated and opposite shoulders were assessed before surgery, intraoperatively and after a minimum two-year follow-up, with special regard to external rotation in adduction (AddER) and abduction (AbdER). Besides, the Constant-Murley score and Subjective Shoulder Value (SSV) were evaluated before a plate removal and after a minimum two-year follow-up after the surgery.

**Results:**

Deltoid adhesion release correlated with considerable and statistically significant improvement of AddER (*p* < 0.0002) but not with the intraoperative range of AbdER. Significant improvement of AddER, but also of AbdER and other range of motion was noted at the follow-up. The improvement of the affected shoulder function following arthroscopic plate removal was considerable and statistically significant according to the modified Constant-Murley score (*p* < 0,01) and SSV (*p* < 0.0000) after a minimum of two-year follow-up.

**Conclusions:**

Our findings are the first to highlight the influence of deltoid muscle, plate, and humerus adhesions on limiting external rotation in adduction after ORIF treatment of proximal humerus fractures. These observations allow the identification of a new shoulder evaluation symptom: Selective Glenohumeral External Rotation Deficit (SGERD) as well as functional deltohumeral space.

## Background

**Fig. 1:**
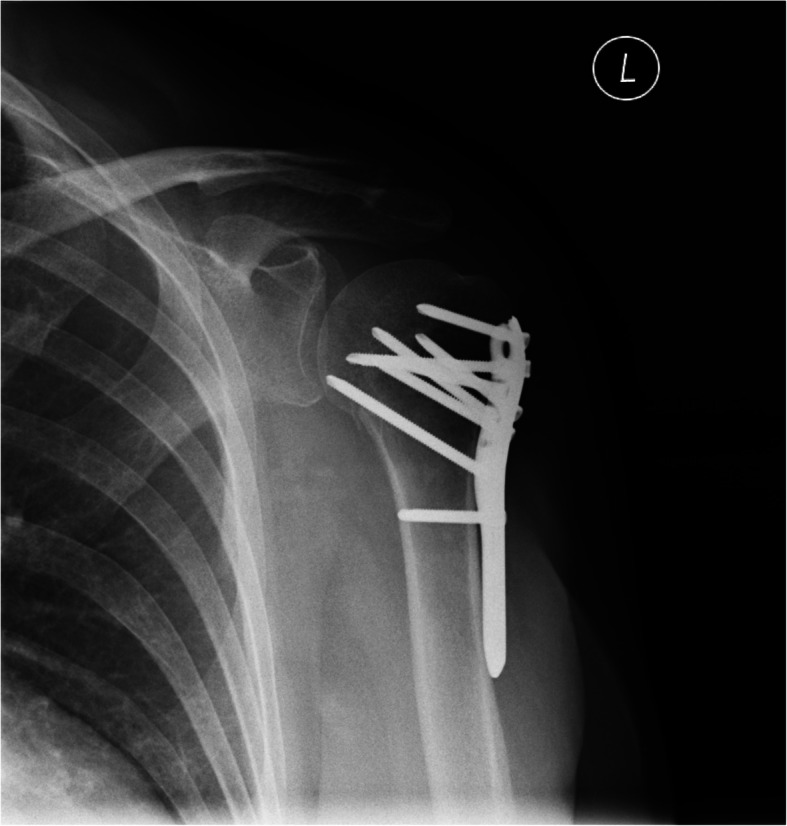
Philos plate before removal (X-ray)

Fractures of the humerus head affect 6% of the elderly population and represent the third most common type of fracture in this age group [1–3]. It is assumed that about 20% of people experiencing such fractures receive surgical treatment [4, 5]. One recognized treatment method is ORIF (Open Reduction and Internal Fixation) using plates (Fig. 1), particularly for multi-fragmental fractures [6, 7]. It is estimated that about 6–30% of patients undergoing ORIF, as a consequence, require removal of the plate due to complications [8–10], the most common causes of which include implant-related irritation, plate destabilization, pain, and limitation of the shoulder range of movement (ROM) [8–12]. The implant removals have been required in 50.6% of cases after minimally-invasive plate osteosynthesis (MIPO); of these, 67.5% of cases were due to an implant-related irritation [1].

A few studies have discussed the possibility of the minimally-invasive removal of plates by arthroscopy [9, 13–17]. However, this is not an easy procedure, especially in the case of MIPO [18]. A key stage of the surgical technique involves determining the course of the axillary nerve concerning the plate, which depends on its location and the height of the patient [19]. Besides, successful intraoperative positioning of the axillary nerve requires its distance from the acromion to be estimated [20–25]. Although this is a straightforward technique, it can be hindered by significant inaccuracy stemming from the variation and dimension of the proximal end of the humerus, the degree of muscularity, and subcutaneous fat content [19]. Although many works confirm that the ROM of the shoulder improves after open or arthroscopic removal of the plate, none discuss the role of adhesions between the deltoid and the plate, or the proximal end of the humerus, particularly concerning their potential impact on the range of motion, especially the external rotation of the shoulder set at adduction [8, 9].

With this in mind, the present study has been conducted to determine how arthroscopic release of deltoid muscle adhesions from the Philos plate and proximal humerus influences the range of external rotation of the shoulder and the subjective improvement of the shoulder function.

## Methods

This study included 18 patients, mean age 56.6 years (23–75 years), treated with ORIF due to a fracture of the proximal humerus. (Table 1, Fig. 1). The indication for arthroscopic plate removal in most cases (*n* = 16) was implant-related irritation, expressed as discomfort combined with post-exertion pain, especially during movement requiring external rotation. The external rotation itself was severely limited (< 20°) in all cases when the arm was placed in adduction (AddER). Impingement was noted between the upper end of the plate with the acromion in two patients (the position of the proximal border of the plate above the humeral head). Two other patients also needed an urgent MRI of the head area due to neurological indications (chronic headache). Patients with severe neurological disorders, hemiplegia, peripheral nerve palsy or brachial plexus injury, failure of the fracture fixation, plate or screws breakage, infection, or avascular necrosis of humeral head were excluded from the study. A total of 14 patients were treated in the Department of Arthroscopy, Minimally Invasive Surgery and Sports Traumatology, Medical University of Lodz (Poland), and the remaining four in the FMC Medical Center (Lodz, Poland) at least 12 months (13–32 months; mean 15.1 ± 5.7 months) after surgery. The primary surgeries (ORIF) were done by the senior author (JF) in 16 cases. Two other cases (in which subacromial impingement syndrome was found) were done in different institution. Plate removal were done by the senior author (JF) in all cases.

**Table 1:** The assessment of range of motion: external rotation in adduction (AddER), external rotation in abduction (AbdER), forward flexion (FFLX), abduction (ABD) and internal rotation (IR) of the operated and healthy shoulder before surgery and after a minimum two-year follow-up. Add/Abd ERI: the adduction-abduction external rotation index. Modified Constan-Murlay score (C-M – 0-75). SSV: Subjective Shoulder Value – before and after plate removal

Factor	Value
No. of patients (shoulder)	18
Dominant/nondominant	10/8
Avarage age (years)	56,6 (23 to 75)
Sex (female/men)	11/7
ROM - range of motion *(°)(*^*a*^*)*	
Operated shoulder, before plate removal	
AddER	7.1 (0 to 15)
AbdER	69.2 (55 to 85)
FFLX	117.8 (104 to 129)
ABD	87.2 (69 to 105)
IR	L2 (L5 to Th9)
Operated shoulder, after plate removal	
AddER	60.3 (45 to 72)
AbdER	78.2 (60 to 90)
FFLX	151.8 (131 to 167)
vABD	128.4 (110 to 142)
vIR	Th11 (L3 to Th8)
Healthy shoulder	
AddER	81 (70 to 90)
AbdER	89.3 (78 to 95)
FFLX	174.6 (168 to 180)
ABD	151.8 (146 to 157)
IR	Th9 (L1 to Th7)
Add/Abd ERI before plate removal	0.11 (0 to 0.19)
Add/Abd ERI after plate removal	0.76 (0.61 to 0.93)
Add/Abd ERI healthy shoulder	0.91 (0.85 to 0.99)
C-M score (0–75) before plate removal	43.2 (35 to 56)
C-M score (0–75) after plate removal	69.4 (62 to 75)
C-M score (0–75) healthy shoulder	72.8 (69 to 75)
SSV before plate removal (%)(^b^)	61.1 (45 to 75)
SSV after plate removal (%)(^b^)	92.8 (80 to 100)

^a^The values in the table (in degrees) are mean ones. They were calculated within a range of extreme values provided in parentheses. ^b^The values in the table (in percentages) are mean ones. They were calculated within a range of extreme values provided in parentheses

**Fig. 2:**
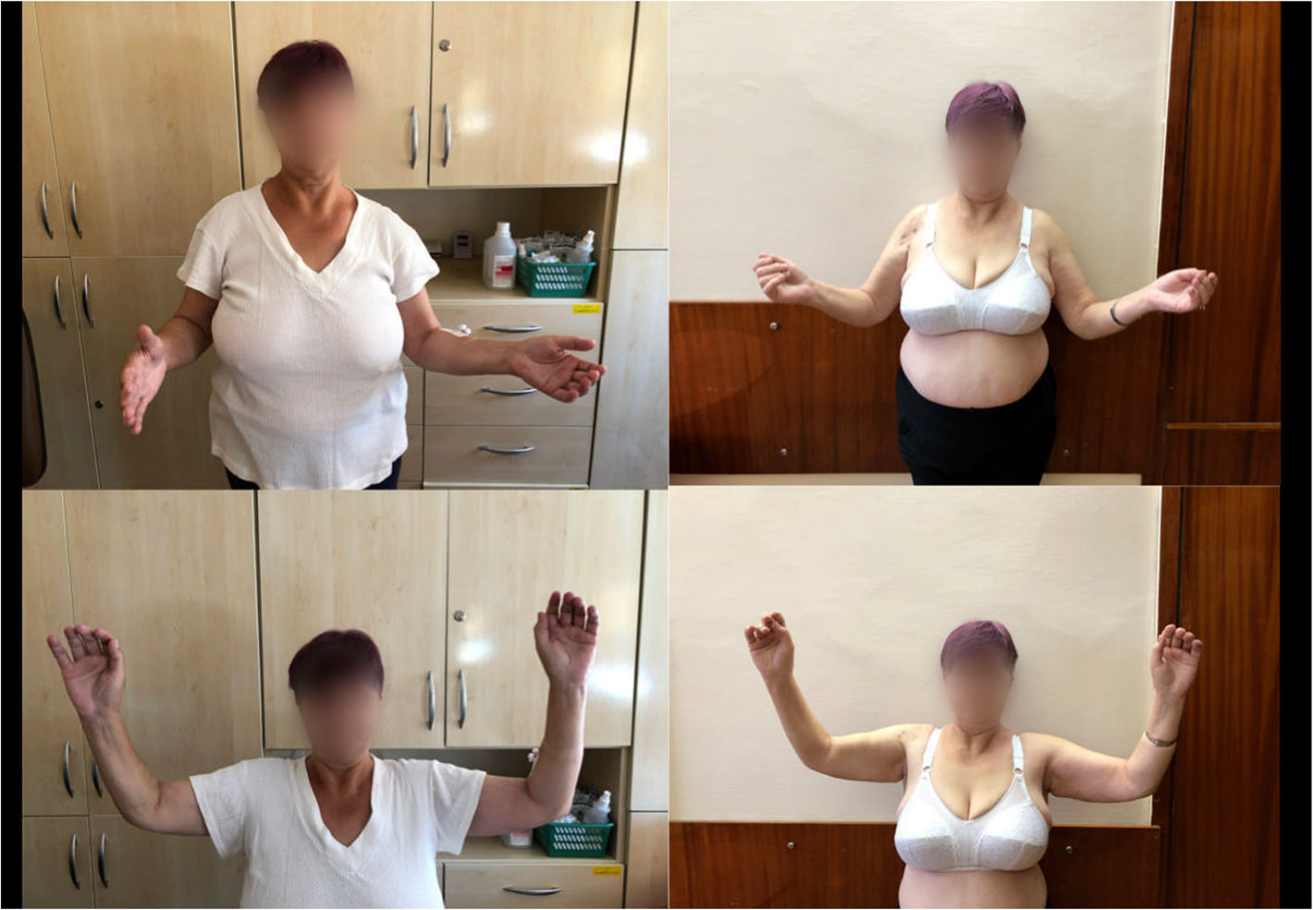
Pre- (*on the left*) and postoperative (*on the right*) ROM assessment: A - Abduction external rotation (AbdER); B - Adduction external rotation (AddER)

The study included a clinical examination of the operated shoulder and the opposite one. The range of motion in all planes was evaluated: forward flexion (FFLX), abduction (ABD), internal rotation (IR), and external rotation (ER). Particular attention was paid to the range of external rotation when the arm was placed in adduction (AddER) and at 90° of abduction in the scapular plane (AbdER) (Fig. 2). The function of both the operated and opposite shoulders was evaluated according to a modified version of the Constant-Murley Score (0–75 points, excluding muscular strength assessment). The strength of the affected shoulder in the abduction was not assessed preoperatively, because the patients were not able to perform active 90° abduction of the shoulder in most cases. Despite the lack of such a measurement, the Constant scale does not lose value, and statistical analysis of comparative data is objective.

Additionally, the subjective shoulder value index (SSV), expressed as a percentage of the value for an entirely normal shoulder (100%), was used to assess the shoulder function [26]. These parameters were assessed before arthroscopic plate removal and again at least 24 months after its removal (24–73 months; mean 38,94 ± 15,95). The range of motion of the operated shoulder was also reassessed intraoperatively by the goniometer. Thanks to intraoperative control of the range of motion, it was possible to determine precisely which part of the procedure – a release of adhesions of the subacromial space to the extent necessary to expose the proximal border of the plate, or a release of adhesions between the muscle, plate, and proximal humerus and plate removal affect the change of external rotation in adduction and abduction. All patients gave informed and written consent to participate in the study. The local Bioethics Committee (Medical University of Lodz, Poland) accepted and approved the study (consent number: RNN /61/ 07 KB), which was conducted following the Helsinki Declaration.

### Statistical analysis

The statistical analysis was performed using Statistica 13.1 PL software (StatSoft Poland, 2013). The Shapiro-Wilk test was used to determine whether the data were normally distributed, with the Student’s t-test or Wilcoxon test used based on the result. *P*-values < 0.05 were regarded as significant. Additionally, the percentage of external rotation deficit (i.e. the quotient of the external rotation of the operated side to that of the controlled healthy side) was calculated for AddER and AbdER before plate removal. Finally, the adduction-abduction external rotational index (Add/Abd ERI) was calculated, i.e. the quotient of the percentage of external rotation deficit in adduction to the percentage of external rotation deficit in the abduction.

### Arthroscopic procedure

**Fig. 3:**
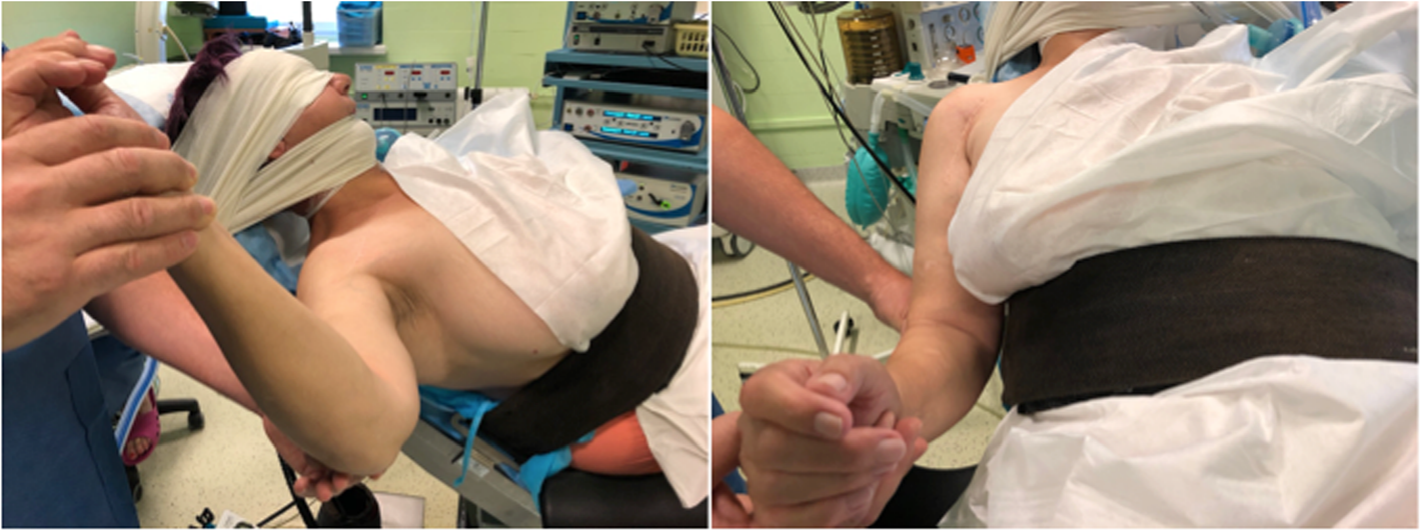
Beach chair position and ROM assessment before surgery. A – AbdER; B – AddER

The patient was placed in a beach chair position and the procedures were performed under general anesthesia. After the onset of anesthesia, the range of motion of the operated and the opposite shoulders was assessed once again (Fig. 3). To open the subacromial space of the shoulder and to isolate the upper pole of the plates, the preliminary traction ranging from 3 to 6 kg was used.

### Portals and adhesions removal

The operation began with the creation of standard posterior and lateral portals. From the posterior portal, the adhesion of the subacromial space was gently released with a trocar in the lateral-anterior direction; following this, a shaver and an electrocauter were introduced from the lateral portal to further release the adhesions and perform a partial bursectomy to allow visualization of the cranial part of the plate. In the next part of the procedure, the traction was released, and external rotation in adduction and abduction was evaluated. Subsequently, the adhesions between the plate and the deltoid muscle were additionally released using an arthroscopic trocar, shaver, and electrocautery. During this stage, the active part of the shaver and the tip of the electrocautery must be in constant contact with the plate to avoid damage to the axillary nerve. Further information about the position of the nerve can be obtained by noting visible contractions of the deltoid muscle during the use of the electrocautery, which was recorded in five cases. After the deltoid adhesion, the range of motion of the operated shoulder was assessed once again.

**Fig. 4:**
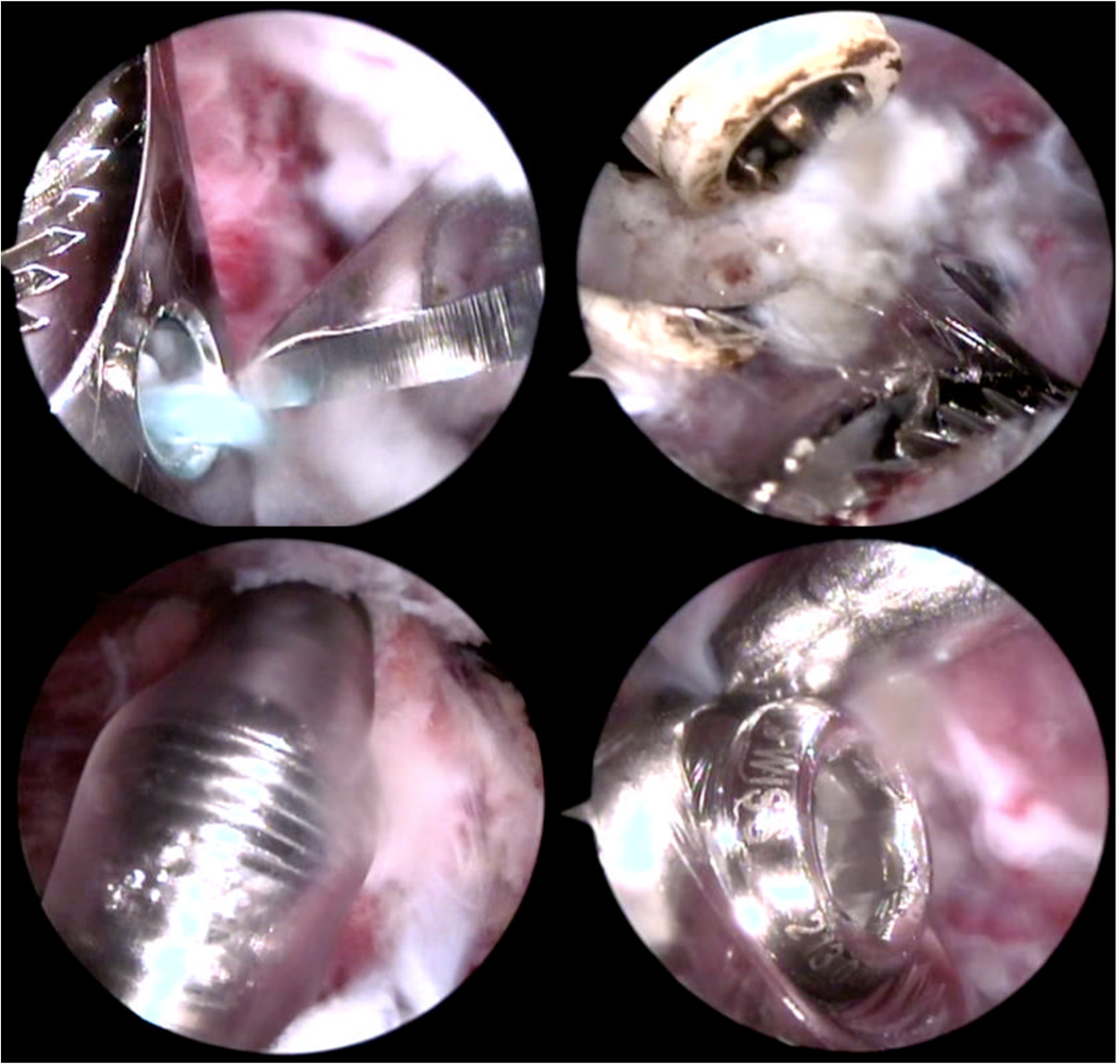
Arthroscopic view of Philos plate and screw removal with the use of a screwdriver and Redon’s drain 18

**Fig. 5:**
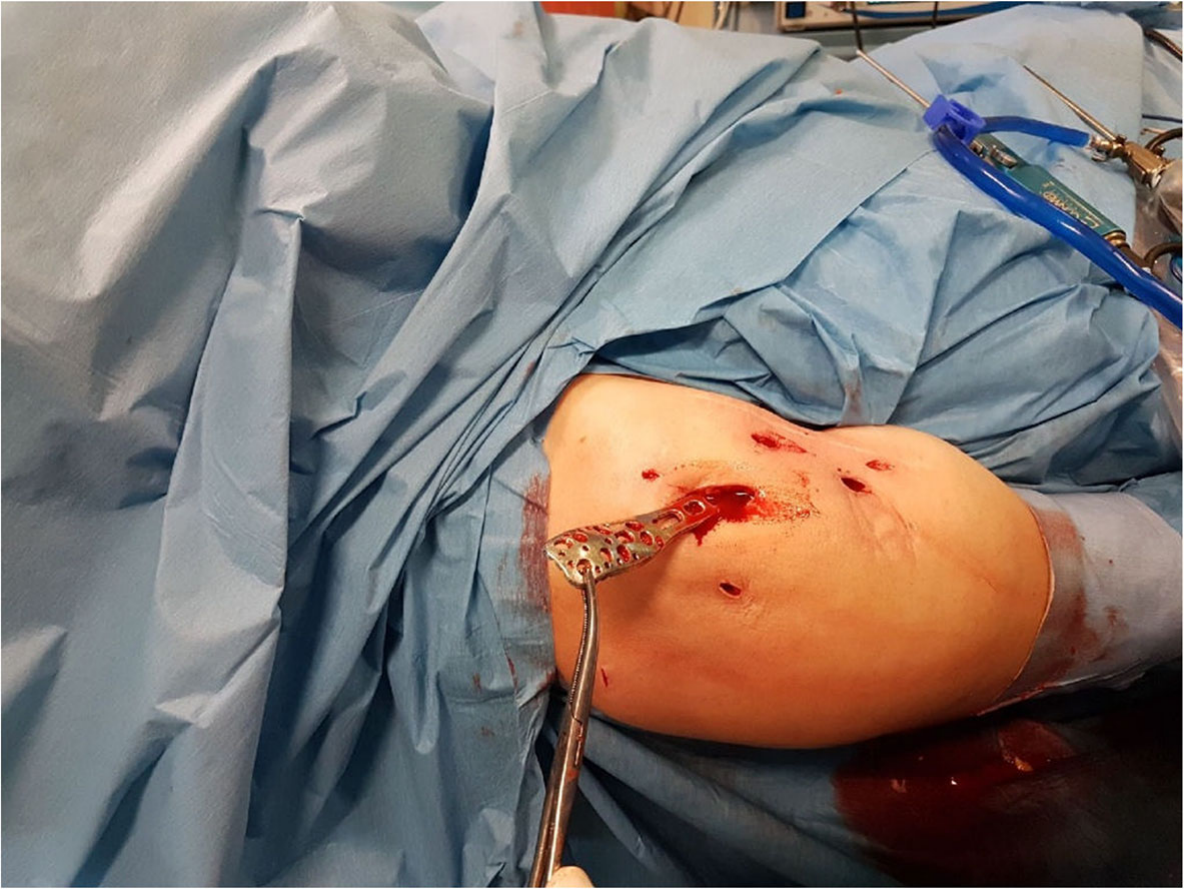
Philos plate removal and additional portals

**Fig. 6:**
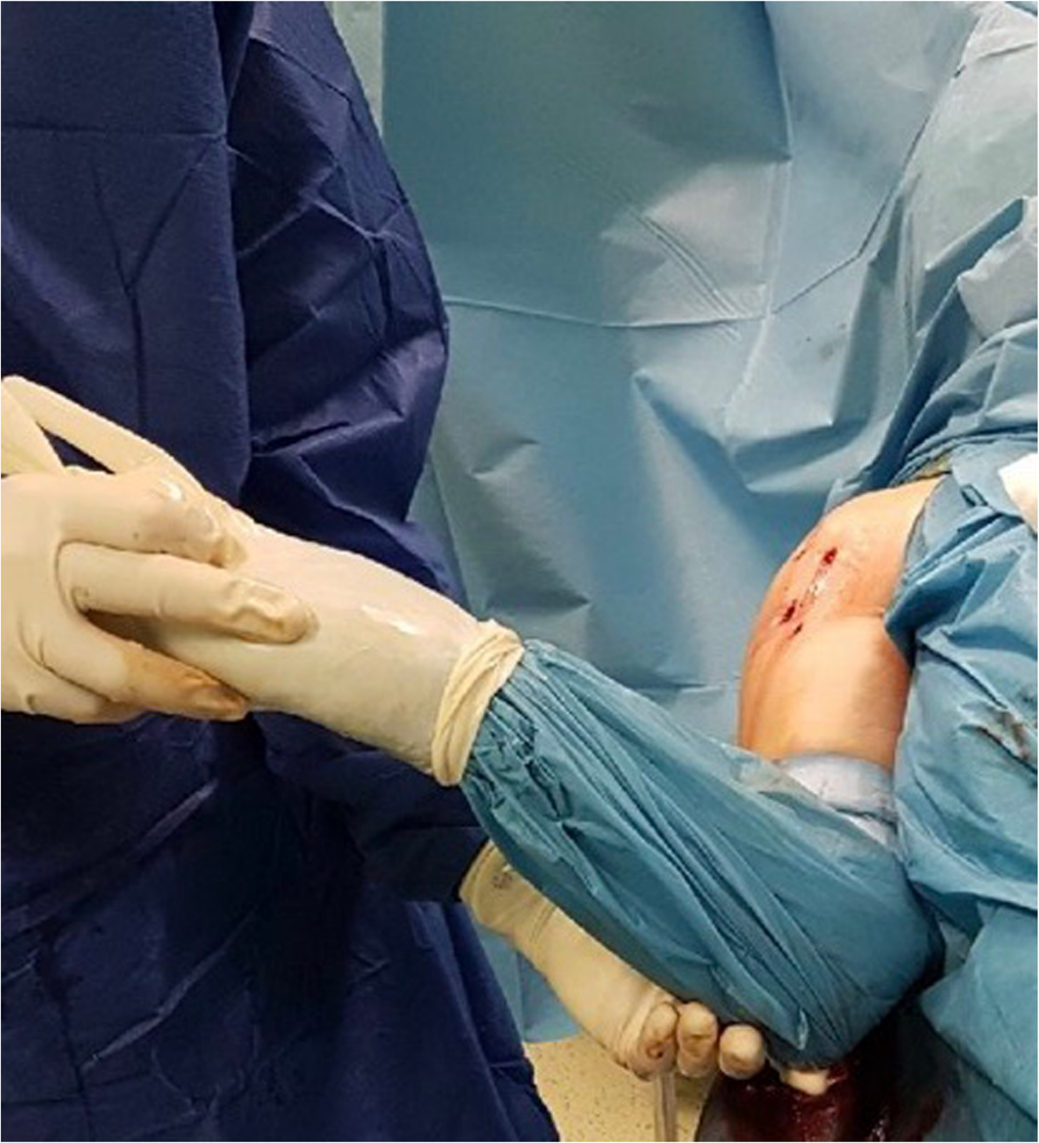
ROM (AddER) assessment after plate removal

To remove the screws, further portals were formed over the long arm of the plate and their locations were determined using a puncture needle, taking into account the course of the axillary nerve. The sutures fixing the tubercles, or the humeral head were excised with an arthroscopic knife and removed with a grasper (Fig. 4). To prevent the damage to the screw head, the axial insertion of the screwdriver into the screw head was supported by the proper arm position (rotation). Typically, all screws in the proximal part of the plate were removed through the anterolateral portal (Fig. 5). In seven patients, one to three screws from the wider part of the plate lost their threading; these were removed with a grasper inserted into an additional portal created with a puncture needle. Finally, after dissecting the plate it and levering it with a rasp, the plate was removed via the lateral portal, which had been widened to about 2 cm (Fig. 5). At the end of the procedure, the range of motion of the operated shoulder was reassessed (Fig. 6).

### Post-operative treatment and rehabilitation

Rehabilitation of the operated shoulder begun on the day of surgery. The first phase of the rehabilitation protocol involved passive and active shoulder movements in all planes. Particular attention was paid to obtain the ranges of motion that were achieved intraoperatively. As the pain subsided, progressive passive, active, and resisted exercises were continued for the next 12 weeks.

## Results

After a minimum of 12 months following ORIF, in all 18 patients included in the study, the range of motion (ROM) of the operated and opposite shoulders were assessed. In all patients, the operated shoulder displayed a reduced ROM relative to the opposite side: the mean degree of FFLX was 117.8°, ABD was 87.2°, IR – L2 spinous process, AbdER was 69.2° and AddER 7.1° (Table 1). The release of adhesions of the subacromial space did not affect AbdER or AddER intraoperatively: improvement in AddER occurred only after the later release of the deltoid muscle adhesion (Table 1). There was no intraoperative improvement of AbdER after deltoid adhesion. However, the gradual improvement was observed at the time of the follow-up (*p* < 0.0002 Wilcoxon test) (Table 1). Significant differences were found between the control shoulder and the operated shoulder concerning AddER (*p <* 0.0002, Student’s T-test) and AbdER (*p <* 0.0002 Wilcoxon test). Further analysis showed that before the release of deltoid adhesions, AddER demonstrated a significantly greater percentage reduction than AbdER, compared to the control shoulder (*p <* 0.0002, Student’s t-test; Table 1). The mean value of the AddER/AbdER index before plate removal was 0.11. There were no statistical differences between the intraoperative range of motion and the postoperative range of motion after a minimum of two-year follow-up.

The function of the affected shoulder improved considerably and statistically significantly following arthroscopic plate removal according to the modified version of the Constant-Murley score (0–75) after a minimum of two-year follow-up (from 43.2 before plate removal to 69.4 after the surgery; *p* < 0,05). The detailed data are presented in Table 1.

In addition, a significant improvement was observed in SSV (from 61.1% before plate removal to 92.8% after plate removal; t-test; *p* < 0.0000, Table 1). None of the patients suffered from damage to the axillary nerve after arthroscopic plate removal.

## Discussion

The key value of our work is confirmation of the selective limitation of external shoulder rotation by adhesion between deltoid, plate, and humerus, which was confirmed intraoperatively with the arm set in adduction. Based on these findings, it has been possible to identify a new shoulder evaluation symptom: Selective Glenohumeral External Rotation Deficit (SGERD).

In all cases reported in this study, the plate was removed at least 12 months after ORIF, which is in agreement with the literature [8, 18]. However, some studies indicate that further improvement of AbdER may be expected even after 2 years from surgery [27]. Moreover, our own experience with the removal of the Philos plates suggests that the number of screws used to fix the implant must also be taken into consideration. Our current preference is to implant as few screws as possible in the long part of the plate (Fig. 1). This approach reduces the length of the surgical incision and the degree of scar tissue formation between the deltoid, plate, and humerus, without compromising the rotational stability or early initiation of careful rehabilitation. However, at least 2 screws should penetrate the cortex to reach the distal fragment (Fig.1).

Limitation of range of motion is quite common after ORIF, and typically correlates with the severity of the fracture [6, 7, 11, 12]; it is also known to occur after conservative treatment [28]. Our own experience confirms the effectiveness of the arthroscopic technique in removing Philos plates and is in line with minimizing surgical trauma [29–31]. No complications associated with axillary nerve injury were observed, both in literature and in our study [19, 22–24, 31]. In all the operated patients, the operated shoulder demonstrated a statistically significant improvement in AddER range of motion (Table 1, Fig. 2, Fig. 6). The clinical significance of the external rotation of the shoulder with the arm set in adduction (AddER) is underestimated since its deficit can be compensated by rotating the trunk and /or changing the position of the whole body. Only the American Shoulder and Elbow Surgeon Scale takes AddER into consideration, its value has no impact on the final evaluation of treatment outcome [32]. However, Warner et al. emphasize the importance of AddER improvement after arthroscopic release for chronic refractory adhesive capsulitis [33].

Implant-related complications, such as irritation, are relatively common and require the removal of the implant [8, 18, 34–37]. Improvements in shoulder function have been observed following implant removal in cases of proximal humerus fracture at the shoulder girdle [8, 18]. Improvements have also been recorded after the removal of an intramedullary rod or plate following midshaft clavicle fracture osteosynthesis; in this case, indications for removal applied to 82% of all cases, regarding the rod, and 50%, regarding the plate [35]. Our findings regarding ROM and SSV index correlating with the Constant score, a valid measure of shoulder function [26], are in agreement with those of previous studies indicating improvement of shoulder function after open or arthroscopic plate removal [8, 9, 13, 16, 18, 38]. However, in contrast to previous publications, our results indicate a new direct correlation between the presence of deltoid muscle, plate and humerus adhesions, and AddER deficit following ORIF treatment of proximal humerus fractures using angular-stable plates.

Interestingly, SGERD could be considered the reverse of GIRD [39, 40] in terms of motion, while internal rotation is limited for shoulder abduction at 90° and no such problem is observed in adduction for GIRD, the opposite is true for SGERD [40]. Our observations might indicate that AddER is typically limited due to the presence of adhesions between the anterior as well as the lateral head of the deltoid muscle, the plate, and proximal humerus; they restrict the natural movement of the humerus in relation to the deltoid muscle. Curiously enough, these adhesions do not affect any restriction of AbdER, which is limited by contracture of the joint capsule, coracohumeral, middle, and inferior glenohumeral ligaments [33, 41–43]. The phenomenon occurs in response to abduction in the scapular plane, which brings the points of the deltoid muscle attachments closer and neutralizes the impact of its adhesions on AbdER.

Basing on our research and observations of other open procedures performed according to the deltopectoral approach, it can be assumed that this SGERD phenomenon is more frequent than one might expect. In this case, the AddER/AbdER index can serve as a valuable indicator of deltoid adhesions: a value lower than 0.15 may indicate the occurrence of severe SGERD.

Our work, however, has some limitations. Firstly, this group of patients was not very large. Nevertheless, the group eligible for this study was homogeneous in terms of the degree of preoperative limitation of AddER and uniform sequential operative technique combined with multiple intraoperative assessments of the range of motion. Secondly, no full post-operative normalization of AddER was performed; while a capsulo-ligamentous contracture may influence AddER, its limitation is still significantly affected by deltoid adhesions. Further studies on SGERD in patients after other shoulder procedures (e.g. prosthesis fitting) also seem to justify, this symptom in a more reliable and useful way.

## Conclusions

1. The presence of adhesions between the deltoid muscle, the plate, and the proximal humerus might result in a selective limitation of the external rotation of the shoulder with the arm set at adduction (AddER); this could be determined by the selective glenohumeral external rotation deficit of the shoulder (SGERD).

2. Arthroscopic release of the adhesions between the deltoid muscle, proximal humerus and the angular plate stabilizing the proximal end of the humerus allows for a significant improvement in external rotation of the shoulder with the arm set in adduction (AddER), as well as in Constant-Murley score and Subjective Shoulder Value (SSV).

3. The deltohumeral space should be recognized as an important separate functional component of a shoulder girdle.

## Data Availability

The datasets used and/or analyzed during the current study are available from the corresponding author on a reasonable request.

## Abbreviations

ORIF: Open reduction internal fixation
ROM: A range of motion
AddER: Adduction external rotation
AbdER: Abduction external rotation
C-M: Constant-Murley score
SSV: Subjective Shoulder Value
SGERD: Selective Glenohumeral External Rotation Deficit
MIPO: Minimally-invasive plate osteosynthesis
GIRD: Glenohumeral internal rotation deficit
